# A psychometric analysis of the Stress Management Competency Indicator Tool

**DOI:** 10.1093/occmed/kqaf028

**Published:** 2025-05-27

**Authors:** T Dulal-Arthur, J Hassard, W Wang, J Yarker, L Thomson, H Blake

**Affiliations:** Schools of Medicine & Health Sciences, University of Nottingham, Nottingham, UK, NG7 2UH; Queen’s Business School, Queen's University Belfast, Belfast, UK, BT9 5EE; Queen’s Business School, Queen's University Belfast, Belfast, UK, BT9 5EE; Schools of Medicine & Health Sciences, University of Nottingham, Nottingham, UK, NG7 2UH; Affinity Health at Work, London, UK, SW12 9NW; Business School, Birbeck University of London, London, UK, WC1E 7HX; Schools of Medicine & Health Sciences, University of Nottingham, Nottingham, UK, NG7 2UH; Schools of Medicine & Health Sciences, University of Nottingham, Nottingham, UK, NG7 2UH

## Abstract

**Background:**

Work-related stress is a growing concern in the UK, negatively impacting both employee well-being and organizational effectiveness. Addressing work-related stress through effective managerial practices is essential, however, existing tools are often lengthy and impractical for routine use.

**Aims:**

The study aimed to develop and validate a shortened version of the 36-item Stress Management Competency Indicator Tool (SMCIT) to enhance its practicality and relevance to changing working practices, while maintaining reliability and validity in assessing stress management competencies.

**Methods:**

A secondary data analysis was undertaken using data from 224 line managers across 12 organizations. Principle component analysis was applied to reduce the SMCIT from 36 to 19 items. Psychometric analysis, including Cronbach’s alpha, factor loadings and total variance explained, was used to ensure the shortened tool retained its measurement rigour.

**Results:**

The 19-item SMCIT retained four latent constructs: respectful/ responsible, managing the individual within the team, conflict/problem management and organizational resources. The revised tool explained 46% of the variance, compared to 37% for the original, and showed factor loadings ranging from point 0.43 to 0.86. Reliability scores ranged from 0.65 to 0.69, indicating moderate internal consistency. We conclude that the shortened tool enhances measurement efficiency by removing redundant items, while maintaining key competencies.

**Conclusions:**

The shortened SMCIT is a reliable and practical tool for evaluating line management competencies, reducing response burden while improving data quality and relevance to hybrid working. We recommend further validation through confirmatory factor analysis and expert review to enhance its application in occupational settings.

## Teaser

Work-related stress presents a challenge to employee well-being and organizational effectiveness. This study undertakes a psychometric evaluation of the Stress Management Competency Indicator Tool (SMCIT), reducing it from 36 to 19 items. The refined tool enhances practical utility and measurement efficiency, facilitating the assessment of managerial competencies in stress management. Findings underscore its potential for integration into organizational interventions aimed at mitigating occupational stress and promoting a healthier work environment.

## INTRODUCTION

Work-related stress is a concern for employers, organizations and society at large, due to its profound and negative impact on individual health [1] and organizational effectiveness [2]. Work-related stress refers to the ‘harmful reaction people have to undue pressures and demands placed on them at work’ [3]. In the United Kingdom (UK), the prevalence of work-related stress is rising; Labour Force Survey estimates suggest that 2290 per 100,000 workers experienced work-related stress, anxiety or depression in 2023/24 resulting in 16.4 million days lost [4].

Work-related stress is linked to adverse mental and physical health outcomes [5]. A systematic review examining the effects of work-related stress among young workers found a significant, negative effect on mental health [6]. At the organizational level, conditions such as work-related anxiety and depression can increase the risk of sickness absenteeism [7], lead to higher turnover intentions [8], impact team dynamics [9] and engender counterproductive workplace behaviours [10]. In the UK, work-related stress and burnout cost the economy around £28 billion annually, resulting in ~ 23.3 million sick days [11].

Recent policy initiatives—such as the Mental Health at Work Guidance [12] and the ISO 45003 Standards on Psychological Health and Safety at Work [13]—promote employee mental well-being by emphasizing the importance of cultivating an enhanced psychosocial working environment through organizational-level interventions including improved line manager training, which is increasingly offered by organizations [14]. Managers play an important role in creating health-promoting physically safe work environments and are strategically positioned to buffer the negative impacts of job demands on their employees through how they manage and organize their team’s work [15]. Their influence extends beyond daily management as line manager training in mental health is linked to adopting other positive mental health and wellbeing practices [16], reduced presenteeism [17] and improved business outcomes [18].

Hence, the enhancement of an organization’s occupational health strategy should comprise developing managers’ competencies in preventing, controlling and managing stress among those they manage (their direct reports [19]). The Health and Safety Executive (HSE) and Chartered Institute of Personnel and Development (CIPD) developed the Stress Management Competency Indicator Tool (SMCIT) to assess managers’ effectiveness at preventing and reducing work-related stress among their staff [20]. As a self-assessment measure, the tool allows managers to consider a range of behaviours that have been identified as important to worker’s work-related stress. Managers’ responses are scored to assess their stress management competencies. The original version of the SMCIT consists of 66 items measuring four management competencies: (1) managing emotions and having integrity, (2) managing and communicating existing and future work, (3) managing the individual within the team and (4) reasoning and managing difficult situations. Each competency comprises three subdivisions reflective of key management behaviours and capabilities, representing 12 sub-competencies (see Table 1).

**Table 1.** SMCIT: competencies, sub-competencies and definitions

Competencies	Definition	Sub-competencies and definitions
Being Respectful/Responsible	Involves behaviours that uphold ethical standards, regulate emotions and display empathy.	**Integrity.** Being honest, respectful and ethical in interactions with employees. **Managing emotions.** Remaining composed and consistent in demeanour, even in challenging situations. **Considerate approach.** Thoughtfully delegating tasks and managing others with sensitivity.
Managing and communicating existing and future work	Includes competencies for proactive work management, effective problem-solving and participatory leadership.	**Proactive work management.** Prioritizing tasks, reviewing work and planning future projects. **Problem-solving.** Swiftly and efficiently addressing issues as they arise. **Participative/empowering.** Involving team members in decisions and empowering them with autonomy.
Managing the individual within the team	Relates to accessibility, sociability and empathetic engagement with direct reports.	**Personally accessible.** Being available for personal conversations and discussions. **Sociable.** Promoting open communication through an approachable attitude. **Empathetic engagement.** Actively seeking to understand employees’ perspectives.
Reasoning and managing difficult situations	Involves the skills to manage conflicts, leverage organizational resources and take responsibility for resolution.	**Managing conflict.** Fairly and promptly addressing conflicts within the team. **Use of organizational resources.** Seeking advice when needed. **Taking responsibility.** Approaching issues with a supportive and responsible mentality.

**Key learning points**

**What is already known about this subject:**

- Work-related stress significantly impacts employee well-being and organizational effectiveness.
- Line managers play a crucial role in managing workplace stress, as they directly influence the work environment and employees’ experiences.
- The Stress Management Competency Indicator Tool (SMCIT) is designed to assess line managers’ abilities to effectively manage stress within their teams, but its original length and changes to working practices following the COVID-19 pandemic may limit its practical use.

**What this study adds:**

- This study successfully refined a shorter, 19-item version of the SMCIT, maintaining its conceptual integrity while improving usability.
- The new version of the tool effectively measures stress management competencies, making it a valuable resource for assessing these skills.
- The study highlights the benefits of using a data-driven approach for scale reduction and creates a more efficient and relevant tool without compromising reliability or validity.

**What impact this may have on practice or policy:**

- The shortened 19-item SMCIT can be more easily integrated into practice, for use in measuring the outcomes of organizational interventions.
- Availability of a shorter tool may increase uptake by researchers and practitioners meaning organizations can more easily assess and develop managerial competencies related to stress management.
- The adoption of this tool may encourage wider implementation of stress management practices, which aligns with national and international policies on workplace health.

During development, stakeholders and workshop participants agreed the tool would be useful for policy review, development and leadership training [20]. However, despite its usefulness, the length of the original tool (66 items) limits its practical applicability, warranting consideration for more streamlined alternatives [21]. In response, Toderi and Sarchielli [21] assessed the tool’s psychometric properties and developed a shorter 36-item version of the questionnaire. They conducted a confirmatory factor analysis (*n* = 353) to reduce the number of items, ensuring that each of the four competencies had an equal number of items. The three most representative items were selected, resulting in 36 items. Though the scale maintains its reliability and validity, the limitation of relying solely on a conceptually driven approach is the risk of oversimplifying the data. By not allowing the data to speak for itself, valuable nuances and patterns may remain unexplored [22]. In contrast, using a data-driven technique can recognize new dimensions or groupings not initially evident and can better identify the underlying structure of the competencies [23]. Moreover, further streamlining the 36-item version of the SMCIT has many noteworthy conceptual, practical and empirical benefits.

Assessing the psychometric properties of the SMCIT is crucial due to conceptual limitations in its items. As the SMCIT was created pre-pandemic, its items may reflect outdated standards due to changes in management practices [24]. For example, the item ‘*Prefers to speak to me personally rather than use email’* might lack applicability within a remote work setting. Although video platforms, such as Teams or Zoom, mimic in-person interactions, they often lack the spontaneity, non-verbal cues and depth inherent in in-person communication. As such, the concept of ‘personal’ interaction in this context could be perceived as less achievable, and therefore the item could be less applicable to modern management practices. By evaluating its psychometric properties, irrelevant items can be removed, enhancing the tool’s overall validity in the current context.

Furthermore, longer questionnaires can be taxing for respondents, leading to participant disengagement, rushed responses and increased measurement errors, which can compromise data quality due to the straight lining of responses (i.e. selecting the same options) [25]. Reducing the length of the questionnaire minimizes participant burden, which may decrease the likelihood of response errors, inaccuracies and nonresponse bias [26], while maintaining reliability and predictive validity [26], thereby offering a more effective approach to data collection [27].

This study aims to provide researchers and organizations with a shortened version of the SMCIT which is reliable, valid and user-friendly. This could encourage the uptake of the tool in organizational settings to mitigate and prevent work-related stress by assessing and developing managerial competencies. The research questions (RQ) are: RQ1: ‘What is the optimal number of items needed for the shortened version of the 36-item SMCIT to adequately measure stress management competencies?’ and RQ2: ‘What is the reliability of a shortened version?’

## METHODS

The SMCIT psychometric properties were analysed using a principal component analysis (PCA), performed on secondary data. PCA captures the maximum variance in the observed variables using a smaller number of derived components. This was chosen over exploratory factor analysis which aims to identify common factors that explain the covariation among the observed variables [28], as it is better suited to the goal of data reduction [29]. Several criteria were assessed to ensure data suitability for conducting a PCA. Hair *et al.* [29] recommend that the sample size should exceed 100 and correlations should range from 0.30 to 0.90. The Kaiser–Meyer–Olkin (KMO) test was employed where scores equal to or above 0.50 indicated PCA suitability. Bartlett’s test of sphericity was also examined where significant values confirm data factorability. Four factors were predetermined for extraction, corresponding to the four targeted competencies. Oblique rotation was applied to gauge the degree of factor inter-correlation. Deletion of items was based on (1) when the proportion of items demonstrating multiple loadings on more than one factor exceeded that which was acceptable within Thurstone’s criteria of 0.20 and (2) factor loadings less than 0.4 [30]. When the objective of a PCA is item reduction, an iterative approach is recognized as one of the strongest methodologies [31].

The secondary data was drawn from a multi-site, two-armed clustered randomized control trial testing the feasibility of a new line manager training intervention which was conducted among eight organizational sectors in the UK [32]. The data were collected between July 2021 and June 2022. To be eligible, participants needed to be at least 18 years old, have direct managerial responsibility, access training materials, receive reminders via work devices and have a valid email address. Employees nearing retirement or redundancy in 6 months and those recently trained in mental health were excluded.

The data used in this study represent the baseline information from participants from both the intervention and control arm, totalling 224 line managers. Line managers completed the 36-item version of the SMCIT alongside four other scales and demographics questions. Mundfrom *et al.* [33] explored the minimum sample size required for such exploratory analyses and noted that sample sizes exceeding 200 are generally sufficient for producing stable factor structures, making this sample appropriate for the research objectives.

Organizational consent was sought before participation. Line managers participating in the study provided written informed consent. Participants understood the voluntary nature of their participation and withdrawal consequences (withdrawn data might be used with consent). Unique codes de-identified participants and confidentiality was preserved with restricted access to password-protected web servers. Ethical approval for this study was obtained on [15 July 2021] (Ref: FMHS 299-0621) by the University of Nottingham School of Medicine Ethics Review Committee.

## RESULTS

The sample included 66% women (*n* = 158) and 34% men (*n* = 83). Participants’ mean age was 44.68 years (SD = 8.29) and the mean tenure in a supervisory role was 12.48 years (SD = 7.1). The dataset comprises 26 organizations across 10 different sectors, with the most represented being Education (*n* = 8, 31%) and Other Service Activities (*n* = 7, 27%). Other sectors include Public Administration and Defence (*n* = 2, 8%), Accommodation and Food Service Activities (*n* = 2, 8%) and Human Health and Social Work Activities (*n* = 1, 4%).

Data factorability was assessed initially for suitability. Of concern, some correlations between variables were below the recommended threshold of 0.30. However, the KMO value of 0.79 indicated adequate inter-correlation. Moreover, Bartlett’s test of sphericity was significant (χ^2^ = 2385.32, df = 630, *P* < 0.001), indicating significant variable correlation and supporting data (available as Supplementary data at *Occupational Medicine* online) factorability. Appendix B (available as Supplementary data at *Occupational Medicine* online) provides a correlation matrix of the study variables.

To address RQ1 *‘What is the optimal number of items needed for the shortened version to adequately measure stress management competencies?’*, item-level statistics were analysed, including the mean, standard deviation, item-total correlation, alpha if item deleted and scale alpha. Item means ranged from 3.28 to 4.55 and scale reliabilities ranged from 0.49 to 0.68 (see Table 2). Although alphas were below the recommended threshold of 0.70, removing items could improve reliability, justifying SMCIT’s length reduction.

**Table 2.** Item-level statistics for the 12 sub-competencies

Sub-competencies	Mean	SD	Item-total correlation	Alpha if item deleted	Alpha
**Integrity**					0.52
Integrity 2	4.55	0.51	0.33	0.46	
Integrity 3	4.53	0.52	0.50	0.22	
Integrity 5	3.55	0.87	0.27	0.66	
**Managing emotions**					0.64
M.E 1	3.96	0.72	0.45	0.47	
M.E 2	4.02	0.64	0.455	0.54	
M.E 4	3.78	0.76	0.40	0.61	
**Considerate**					0.49
Considerate 2	4.13	0.62	0.37	0.36	
Considerate 5	4.03	0.65	0.25	0.50	
Considerate 6	4.40	0.55	0.36	0.32	
**Pro-work**					0.51
Pro-work 5	3.91	0.75	0.22	0.58	
Pro-work 8	4.02	0.77	0.37	0.34	
Pro-work 9	3.77	0.72	0.40	0.38	
**Problem-solving**					0.65
P.S 2	4.24	0.58	0.41	0.62	
P.S 3	3.78	0.73	0.53	0.45	
P.S 4	3.84	0.79	0.46	0.56	
**Empowering**					0.57
Empowering 1	3.97	0.53	0.46	0.36	
Empowering 2	3.91	0.64	0.37	0.50	
Empowering 7	4.31	0.55	0.33	0.55	
**Accessible**					0.45
Accessible 1	3.99	0.76	0.13	0.75	
Accessible 3	4.25	0.61	0.48	0.11	
Accessible 4	4.43	0.57	0.39	0.29	
**Sociable**					0.57
Sociable 1	3.48	1.04	0.40	0.45	
Sociable 2	3.28	0.97	0.47	0.31	
Sociable 3	4.32	0.57	0.32	0.57	
**Empathetic engagement**					0.65
E.E 3	4.02	0.69	0.55	0.42	
E.E 4	4.21	0.56	0.44	0.59	
E.E 5	4.01	0.82	0.42	0.63	
**Managing conflict**					0.68
M.C 3	3.89	0.62	0.58	0.49	
M.C 4	3.64	0.79	0.48	0.62	
M.C 1	3.85	0.64	0.44	0.65	
**Org resources**					0.65
O.R 1	4.27	0.70	0.33	0.70	
O.R 2	3.81	0.99	0.59	0.34	
O.R 3	3.61	1.00	0.49	0.51	
**Resolving issues**					0.60
R.I 1	3.70	0.70	0.36	0.56	
R.I 2	3.77	0.75	0.47	0.39	
R.I 3	4.09	0.70	0.38	0.53	

The item removal process was conducted iteratively, eliminating items that did not meet the established criteria until a clean factor structure was obtained. This iterative approach ensured that the remaining items effectively captured the distinct competencies without excessive redundancy or ambiguity. A total of seven iterations were undertaken, with each iteration removing at least one poorly fitted item and one cross-loading. The pattern matrix displayed the factor loadings for each item across the four components, indicating the strength and direction of the relationship between the items and their respective competencies. Table 3 below summarizes the findings of the finalized four-factor solution. We observe that the optimal number of items needed to adequately measure stress management competency is 19. See Appendix A (available as Supplementary data at *Occupational Medicine* online) for a full description of the content for each item.

**Table 3.** Finalized four-factor PCA solution

Items	Components			
	Being Respectful/ Responsible	Managing the individual within the team	Conflict/Problem management	Organizational resources
Integrity 2	0.76			
Integrity 3	0.67			
Integrity 5	0.44			
Managing emotions 1	0.53			
Managing emotions 2	0.59			
Managing emotions 4	0.58			
Problem-solving 2				−0.44
Problem-solving 3				−0.66
Problem-solving 4				−0.81
Sociable 1		0.67		
Sociable 2		0.76		
Sociable 3		0.64		
Empathetic engagement 3		0.50		
Empathetic engagement 5		0.71		
Managing conflict 4				−0.57
Managing conflict 1				−0.55
Organizational resources 1			0.56	
Organizational resources 2			0.86	
Organizational resources 3			0.81	

Table 4 illustrates the shift and consolidation of competencies and sub-competencies as the measure moved from 36 items to 19 items. The ‘Being Respectful/Responsible’ competency was condensed to only retain the sub-competencies of *integrity* and *managing emotions*. Similarly, ‘Managing the individual within the team’ only retained *sociable* and *empathetic engagement*. Contrary to the original scale, *problem-solving* and *managing conflict* loaded together, transforming the macro-competency from ‘Managing and communicating existing and future work’ to ‘Conflict/Problem management’. Finally, *organizational resources* loaded onto itself, shifting the competency of ‘Reasoning and managing difficult situations’ to ‘Organizational resources’.

**Table 4.** Comparison between 36-item version (Toderi and Sarchielli [21]) and 19-item version

Mapping of 36-item version		Mapping of 19-item version	
Competencies	Sub-competencies	Competencies	Sub-competencies
Being respectful/ responsible	Integrity Managing emotions Considerate approach	Respectful/ Responsible	Integrity Managing emotions
Managing the individual within the team	Sociable Empathetic engagement Personally accessible	Managing the individual within the team	Sociable Empathetic engagement
Managing and communicating existing and future work	Proactive work management Problem-solving Participative/empowering	Conflict/Problem management	Problem-solving Managing conflict
Reasoning and managing difficult situations	Managing conflict Use of organizational resources Taking responsibility	Organizational resources	Use of organizational resources

To verify that 19 items are optimal for measuring stress management competencies, the total variance explained by the new scale was examined. Table 5 highlights the progression of total variance explained between the initial 36-item version of the SMCIT and the reduced 19-item counterpart. The cumulative percentage of variance explained increased from 36.54 to 46.06. This indicates a substantial improvement of the latent factors’ explanatory power through the refinement of the scale and further supports the efficacy of the 19-item version to capture the core competencies more succinctly and effectively.

**Table 5.** Explained variance: comparing the 19-item and 36-item SMCIT versions

Component	Total	% of Variance	Cumulative %
1	3.71 **(6.99)**	19.53 **(19.40)**	19.53 **(19.40)**
2	2.03 **(2.23)**	10.67 **(6.20)**	30.20 **(25.60)**
3	1.59 **(2.10)**	8.36 **(5.82)**	38.56 **(31.43)**
4	1.43 **(1.84)**	7.51 **(5.11)**	46.06 **(36.54)**

Note: Values in **bold** signify results from the 36-item version.

Research question two was answered by examining the internal consistency of the scales, captured by Cronbach’s alpha. Table 6 shows that the competencies’ alphas ranged from 0.65 to 0.69—indicating moderate reliability. Similarly, the sub-competencies’ alphas ranged from 0.49 to 0.65—also indicating moderate reliability.

**Table 6.** Reliabilities of the new four-factor solution

Competencies	Reliabilities (Cronbach’s alpha)
Respectful/ responsible	0.65
Managing the individual within the team	0.69
Organizational resources	0.65
Conflict/ Problem management	0.65
**Sub-competencies**	
Integrity	0.52
Managing emotions	0.64
Problem-solving	0.65
Sociable	0.57
Empathetic engagement	0.59
Managing conflict	0.49
Organizational resources	0.65

## DISCUSSION

We analysed the psychometric properties of the 36-item version of the SMCIT with the dual aim of reducing the overall length of the scale whilst maintaining its reliability and validity. The PCA resulted in a 19-item version of the SMCIT which had moderate reliability and high validity. A key strength of the study is the use of a large and diverse sample of line managers, drawn from a wide range of organizations and occupational sectors. This provides a unique dataset that facilitates a broader understanding of line managers’ stress management competencies across different contexts, enhancing the generalisability and relevance of the findings. Furthermore, the use of an iterative approach in PCA ensures robust item reduction and accurate factor extraction, improving the reliability of the findings. However, incorporating data from the direct report perspective would have been ideal to facilitate the comparison of the results between samples, as suggested by Toderi and Sarchielli [21]. This would allow us to identify discrepancies between how managers perceive their own skills, knowledge and behaviour(s) and how employees experience it. Hence, items which appear consistent but do not effectively capture manager behaviour could be refined. Another limitation is that the scale was only analysed using an exploratory approach, which is the initial phase of scale refinement. Though it helps to identify the underlying factor structure, it does not confirm this structure.

Future research should replicate the study with a different sample of managers, using the 19-item version of the SMCIT to validate the scale using a confirmatory approach where key fit indices such as chi-square goodness of fit, RMSEA, CFI and AVE can be thoroughly assessed. The outcomes of this future analysis would provide further insights into the scale’s suitability for continual use among managers. It is also advisable to cross-validate the scale by seeking expert reviews. This could be achieved by consulting a panel of experts in occupational or public health to assess the scale’s relevance and potential improvements [34]. By undertaking these recommended approaches, future research can strengthen the scale’s validity, ultimately enhancing understanding of stress management competency and contributing to the fields of occupational health and public health by supporting interventions that promote employee well-being and reduce work-related stress.

The factor loadings observed in the current sample were higher relative to those reported in previous studies. For example, in the EFA conducted by Yarker *et al.* [20] on the SMCIT, all factor loadings fell below 0.57, indicative of only modest associations. In the current analysis, however, we observed much higher factor loadings which ranged from 0.44 to as high as 0.86. This improvement in factor loadings suggests that the new scale has stronger associations between the items and their respective competencies and better captures their variability [35]. Assessing the internal consistency of the new scale revealed reliabilities ranging from 0.65 to 0.69 across the four competencies. These reliabilities marginally fall below Pallant’s [36] recommended threshold of 0.7 for acceptable internal consistency and are lower compared to that reported by the 36-item version of the SMCIT [21]. These low reliabilities can be attributed to the reduced number of items within the factors, which likely affected the alpha value. However, Czerwinski and Atroszko [37] noted that despite having lower reliabilities, shorter scales sometimes hold equal or greater validities compared to lengthier scales. Moreover, although alpha values below 0.7 are less than ideal, the overall utility of the scale should not be judged solely by internal consistency. Other important criteria such as the scale’s validity, content relevance and practical contribution to the field should also be considered [38].

There are several noteworthy similarities and differences between the obtained factor structure and the original factor structure. Like the original structure, the sub-competencies of ‘Integrity and Managing Emotions’ continued to load onto the same competency ‘Being Respectful/Responsible’ in the reduced scale. Moreover, the sub-competencies of ‘Sociable and Empathetic Engagement’ also loaded onto the same competency ‘Managing Individual within the Team’. The consistent loading of sub-competencies onto the same competencies in both the original and reduced scales provides cross-validation of the SMCIT factor structure. It implies that the underlying structure of the measured construct remains stable across different samples. This adds to the robustness of the factor solution, showing that the identified factors capture distinct competencies.

Managing conflict loaded onto the same factor in the reduced scale. Although different from the original scale, this finding aligns with the conceptual understanding that problem-solving skills and conflict-management abilities are closely related. In fact, some studies suggest that conflict-management ability encompasses the strategy of problem-solving [39]. For instance, the item ‘I deal with problems as soon as they arise’ (representing problem-solving) captures a similar construct as ‘I deal with conflicts head-on’ (representing managing conflicts). The alignment of these two factors not only makes conceptual sense but also offers enhanced practicality through a reduction in redundancy. Lastly, the sub-competency of ‘Organizational Resources’ was found to load independently, indicating that it represents a distinct factor in the reduced scale. The new dominance of this factor may be explained by the need for managers to navigate constant and rapid change post-pandemic [40], requiring managers to draw more regularly and systematically on organizational resources to protect and promote their own and others’ well-being. Including ‘Organizational Resources’ as a separate factor in the reduced scale ensures that the competencies are comprehensively measured and that unique aspects are accounted for.

This study significantly contributes to both research and practice by providing a practical, streamlined scale that can be easily integrated into the evaluation of organizational interventions and broader workplace practices. It offers a user-friendly, reliable and valid self-reflective development tool for managers and leaders to assess their behaviours related to management of work-related stress within their team. This helps to provide insights to enhance personal development and better leadership practices. From a practical standpoint, this tool could be used by human resource (HR) professionals and occupational health specialists in their ongoing efforts to enhance managerial competencies in the prevention and management of work-related stress of direct reports, which could ultimately improve employees’ well-being.

Given its focus on leadership behaviours, this tool may be particularly valuable when embedded within leadership training, coaching or manager development programmes. Furthermore, it should be integrated into workplace intervention to align with existing standards on work-related stress management and mental health in the workplace (e.g. HSE Management Standards, ISO 45003). Future research should explore how the tool can be best implemented alongside such standards and within structured leadership development initiatives.

## Supplementary Material

kqaf028_suppl_Supplementary_Appendix_A

kqaf028_suppl_Supplementary_Appendix_B
