# Supplementary material for: A psychometric analysis of the Stress Management Competency Indicator Tool
Source: Occup Med (Lond). 2025 May 27;75(3-4):188–95. doi: 10.1093/occmed/kqaf028 (PMC12257942; doi:10.1093/occmed/kqaf028)
Supplement: kqaf028_suppl_Supplementary_Appendix_A [file kqaf028_suppl_supplementary_appendix_a.docx]

**Appendix A: Content of New Scale**

| Item Name | Item Label |
| --- | --- |
| Integrity 2 | I treat my team members with respect |
| Integrity 3 | I am honest |
| Integrity 5 | I never speak about team members behind their backs |
| Managing emotions 1 | I act calmly in pressured situations |
| Managing emotions 2 | I take a consistent approach to managing |
| Managing emotions 4 | I don’t pass on my stress to my team |
| Problem solving 2 | I follow up problems on behalf of my team |
| Problem solving 3 | I deal with problems as soon as they arise |
| Problem solving 4 | I am decisive when decision making |
| Sociable 1 | I bring in treats for my team |
| Sociable 2 | I socialise with the team |
| Sociable 3 | I am willing to have a laugh at work |
| Empathetic engagement 3 | I make an effort to find out what motivates my team members at work |
| Empathetic engagement 5 | I take an interest in my team’s life outside work |
| Managing conflict 4 | I deal with conflicts head-on |
| Managing conflict 1 | I act as a mediator in conflict situations |
| Organisational resources 1 | I seek advice from other managers when necessary |
| Organisational resources 2 | I use HR as a resource to help deal with problems |
| Organisational resources 3 | I seek help from occupational health when necessary |
