# Supplementary material for: A psychometric analysis of the Stress Management Competency Indicator Tool
Source: Occup Med (Lond). 2025 May 27;75(3-4):188–95. doi: 10.1093/occmed/kqaf028 (PMC12257942; doi:10.1093/occmed/kqaf028)
Supplement: kqaf028_suppl_Supplementary_Appendix_B [file kqaf028_suppl_supplementary_appendix_b.docx]

## Assumption Testing: Correlation Table

| **Variable** | **1** | **2** | **3** | **4** | **5** | **6** | **7** | **8** | **9** | **10** | **11** | **12** |
| --- | --- | --- | --- | --- | --- | --- | --- | --- | --- | --- | --- | --- |
| 1. Integrity | 1.0 | 0.318** | 0.27** | 0.156* | 0.219** | 0.212** | 0.305** | -0.02 | 0.251** | 0.221** | 0.118 | 0.192** |
| 2. Managing Emotions | 0.318** | 1.0 | 0.228** | 0.191** | 0.274** | 0.322** | 0.297** | 0.075 | 0.183** | 0.32** | 0.157* | 0.242** |
| 3. Considerate Approach | 0.27** | 0.228** | 1.0 | 0.344** | 0.252** | 0.379** | 0.373** | 0.174** | 0.295** | 0.177** | 0.219** | 0.256** |
| 4. Proactive Work Management | 0.156* | 0.191** | 0.344** | 1.0 | 0.36** | 0.347** | 0.234** | 0.063 | 0.225** | 0.149* | 0.34** | 0.358** |
| 5. Problem Solving | 0.219** | 0.274** | 0.252** | 0.36** | 1.0 | 0.448** | 0.352** | 0.144* | 0.271** | 0.347** | 0.15* | 0.315** |
| 6. Empowering | 0.212** | 0.322** | 0.379** | 0.347** | 0.448** | 1.0 | 0.401** | 0.29** | 0.422** | 0.39** | 0.275** | 0.362** |
| 7. Personally Accessible | 0.305** | 0.297** | 0.373** | 0.234** | 0.352** | 0.401** | 1.0 | 0.32** | 0.404** | 0.262** | 0.102 | 0.244** |
| 8. Sociable | -0.02 | 0.075 | 0.174** | 0.063 | 0.144* | 0.29** | 0.32** | 1.0 | 0.43** | 0.074 | 0.158* | 0.165* |
| 9. Empathetic Engagement | 0.251** | 0.183** | 0.295** | 0.225** | 0.271** | 0.422** | 0.404** | 0.43** | 1.0 | 0.313** | 0.224** | 0.369** |
| 10. Managing Conflict | 0.221** | 0.32** | 0.177** | 0.149* | 0.347** | 0.39** | 0.262** | 0.074 | 0.313** | 1.0 | 0.179** | 0.506** |
| 11 Organisational Resources | 0.118 | 0.157* | 0.219** | 0.34** | 0.15* | 0.275** | 0.102 | 0.158* | 0.224** | 0.179** | 1.0 | 0.39** |
| 12 Resolving Issues | 0.192** | 0.242** | 0.256** | 0.358** | 0.315** | 0.362** | 0.244** | 0.165* | 0.369** | 0.506** | 0.39** | 1.0 |

Note: p<.05*, p<.01**, p<.001***
